# *“Koko et les lunettes magiques”*: An educational entertainment tool to prevent parasitic worms and diarrheal diseases in Côte d’Ivoire

**DOI:** 10.1371/journal.pntd.0005839

**Published:** 2017-09-21

**Authors:** Clémence Essé, Véronique A. Koffi, Abel Kouamé, Kouassi Dongo, Richard B. Yapi, Honorine M. Moro, Christiane A. Kouakou, Marta S. Palmeirim, Bassirou Bonfoh, Eliézer K. N’Goran, Jürg Utzinger, Giovanna Raso

**Affiliations:** 1 Unité de Formation et de Recherche des Sciences de l’Homme et de la Société, Université Félix Houphouët-Boigny, Abidjan, Côte d’Ivoire; 2 Département Recherche et Développement, Centre Suisse de Recherches Scientifiques en Côte d’Ivoire, Abidjan, Côte d’Ivoire; 3 Department of Epidemiology and Public Health, Swiss Tropical and Public Health Institute, Basel, Switzerland; 4 University of Basel, Basel, Switzerland; 5 Afrika Toon, Abidjan, Côte d’Ivoire; 6 Unité de Formation et de Recherche Biosciences, Université Félix Houphouët-Boigny, Abidjan, Côte d’Ivoire; Jiangsu Institute of Parasitic Diseases, CHINA

## Abstract

**Background:**

Integrated control programs, emphasizing preventive chemotherapy along with health education, can reduce the incidence of soil-transmitted helminthiasis and schistosomiasis. The aim of this study was to develop an educational animated cartoon to improve school children’s awareness regarding soil-transmitted helminthiasis, diarrheal diseases, and related hygiene practices in Côte d’Ivoire. The key messages included in the cartoon were identified through prior formative research to specifically address local knowledge gaps.

**Methodology:**

In a first step, preliminary research was conducted to assess the knowledge, attitudes, practices, and beliefs of school-aged children regarding parasitic worm infections and hygiene, to identify key health messages to be included in an animated cartoon. Second, an animated cartoon was produced, which included the drafting of the script and story board, and the production of the cartoon’s initial version. Finally, the animated cartoon was pilot tested in eight selected schools and further fine-tuned.

**Principal findings:**

According to the questionnaire results, children believed that the consumption of sweet food, eating without washing their hands, sitting on the floor, and eating spoiled food were the main causes of parasitic worm infections. Abdominal pain, diarrhea, lack of appetite, failure to grow, and general fatigue were mentioned as symptoms of parasitic worm infections. Most of the children knew that they should go to the hospital for treatment if they experienced symptoms of parasitic worm diseases. The animated cartoon titled “*Koko et les lunettes magiques*” was produced by Afrika Toon, in collaboration with a scientific team composed of epidemiologists, civil engineers, and social scientists, and the local school children and teachers. Pilot testing of the animated cartoon revealed that, in the short term, children grasped and kept key messages. Most of the children who were shown the cartoon reported to like it. Acceptance of the animated cartoon was high among children and teachers alike. The messaging was tailored to improve knowledge and practices for prevention of helminthiases and diarrheal diseases through prior identification of knowledge gaps. Integration of such education tools into the school curriculum, along with deworming campaigns, might improve sustainability of control and elimination efforts against helminthiases and diarrheal diseases.

## Author summary

Soil-transmitted helminthiases, schistosomiasis, and diarrhea remain important public health issues in sub-Saharan Africa. Health educational animated cartoons can help raise awareness and improve hygiene practices, and thus contribute to the control and elimination of these diseases. For the development of an educational animated cartoon, we first evaluated the knowledge, attitudes, practices, and beliefs of school-aged children in Côte d’Ivoire regarding soil-transmitted helminthiases, diarrhea, and schistosomiasis in order to identify setting-specific health messages for our animated cartoon. We found that children believed that the consumption of sweet food, eating without washing their hands, sitting on the floor, and eating spoiled food were the main causes of parasitic worm infections. As a next step, the alpha version of the animated cartoon was produced and given the title: “*Koko et les lunettes magiques*”. The animated cartoon was pre-tested in eight schools and further developed. Our study found that children could retain most of the information provided by the animated cartoon. It is suggested that such a tool, integrated into the school curriculum, together with deworming campaigns in Côte d’Ivoire, might improve the sustainability of control and elimination efforts against soil-transmitted helminthiases, diarrhea, and schistosomiasis.

## Introduction

Infections with soil-transmitted helminths (STHs; i.e., *Ascaris lumbricoides*, *Trichuris trichiura*, and hookworm) are among the most common neglected tropical diseases (NTDs) [1,2]. Indeed, more than one billion people are infected with at least one species of STHs [3]. Parasitic worm infections are intimately linked to poverty, such as inadequate sanitation and waste disposal, lack of access to clean water, poor hygiene, and limited access to health care and preventive measures [4,5]. Although reinfection can occur rapidly after treatment [6], the main strategy to control morbidity due to parasitic worm infections is preventive chemotherapy, that is the repeated large-scale administration of anthelmintic drugs to high-risk groups, particularly school-aged children [7]. Importantly, STHs are amongst the many pathogens causing diarrhea, whereas the condition remains a leading driver of mortality and morbidity among children under the age of 5 years worldwide and disproportionally affects those from low- and middle-income countries (LMICs) [8]. Inadequate water and sanitation, suboptimal breastfeeding, zinc and vitamin A deficiency, and lack of access to quality health care and timely and effective treatment with oral rehydration solution are reasons for the high global burden of diarrheal diseases [8].

Although health education and sanitation are two important components of primary health care emphasized by the World Health Organization (WHO) as a basis for the prevention and control of communicable diseases [9], there is still limited application of health education in the control and elimination of parasitic worm infection as part of an integrated strategy [4,10]. It has been demonstrated that integrated control programs that combine preventive chemotherapy with health education for prevention of re-infections, can reduce prevalence and morbidity of STH and schistosomiasis [11–16]. In particular, health education offers opportunities for the community to improve their health by increasing knowledge and skill sets [17].

The potential of educational videos is well established, as they can engage and inform at the same time. This is of importance, particularly for younger school-aged children as their attention span is limited. Videos can display real-life situations that children can readily grasp. It is essential that the messaging of videos is tailored to the context of the target population. Hence, a preliminary assessment of the knowledge, attitudes, practices, and beliefs (KAPB) of a population can allow the identification of appropriate messages to be incorporated into a story that entertains and engages the audience, thus rendering the educational tool popular and effective in different age groups [13].

In Côte d’Ivoire, NTDs are of considerable public health relevance [18–20]. Recent data obtained from a national school-based survey with more than 5,000 children aged 5–16 years revealed that 26% of the children were infected with at least one species of parasitic worms. Hookworm was identified as the predominant STH infection (overall prevalence was 17%) with a fairly homogeneous distribution. The other STH infections (*A*. *lumbricoides* and *T*. *trichiura*) were found with a prevalence of less than 5% each [21].

The aim of the current study was to develop an educational cartoon that might help improve school children’s awareness regarding STHs and diarrheal diseases, and related hygiene practices in Côte d’Ivoire. The study was guided by experiences and lessons from previous investigations in the People’s Republic of China where a health educational video proved useful for lowering the incidence of STH reinfection rates and improving STH case management. In particular, “The magic glasses concept” employed in the educational video proposed by Bieri and colleagues was applied for the current research [12,22].

## Methods

### Ethics statement

Ethical clearance for the study was obtained from the ethics committee of Basel (EKBB; reference no. 300/13) and from the ethics committee of the Ministry of Health and Public Hygiene in Côte d’Ivoire (reference no. 76-MSLS-CNER-dkn). Local community and school authorities were visited and informed about the purpose and procedures of the study before commencement of the field activities. Because this study involved the participation of school-aged children, each of them provided a written informed consent signed by their parents or legal guardians prior to enrollment. Children assented orally. Participation was voluntary and children could withdraw any time without further obligations.

### Study design and area

**Fig 1:**
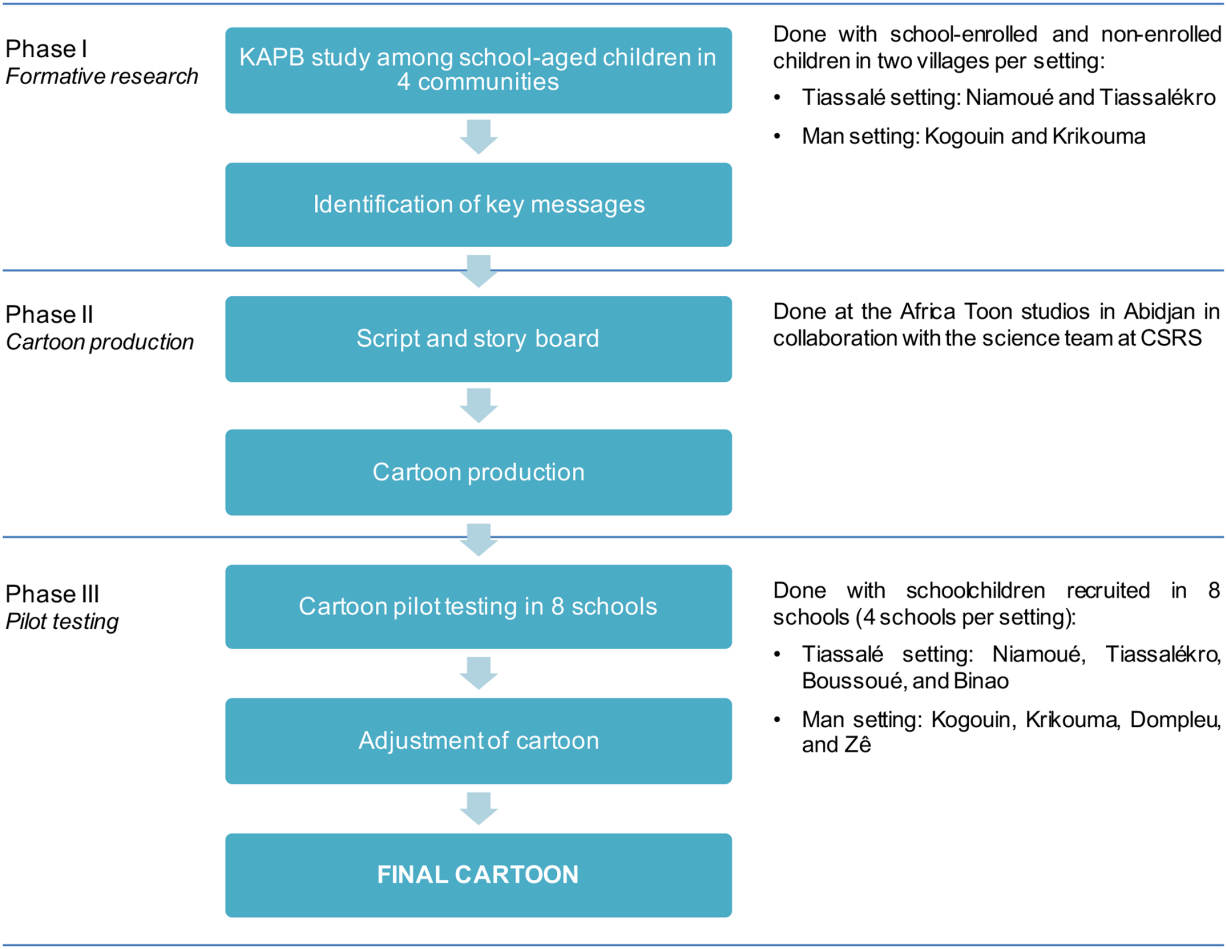
Procedure of “*Koko et les lunettes magiques*” production.

**Fig 2:**
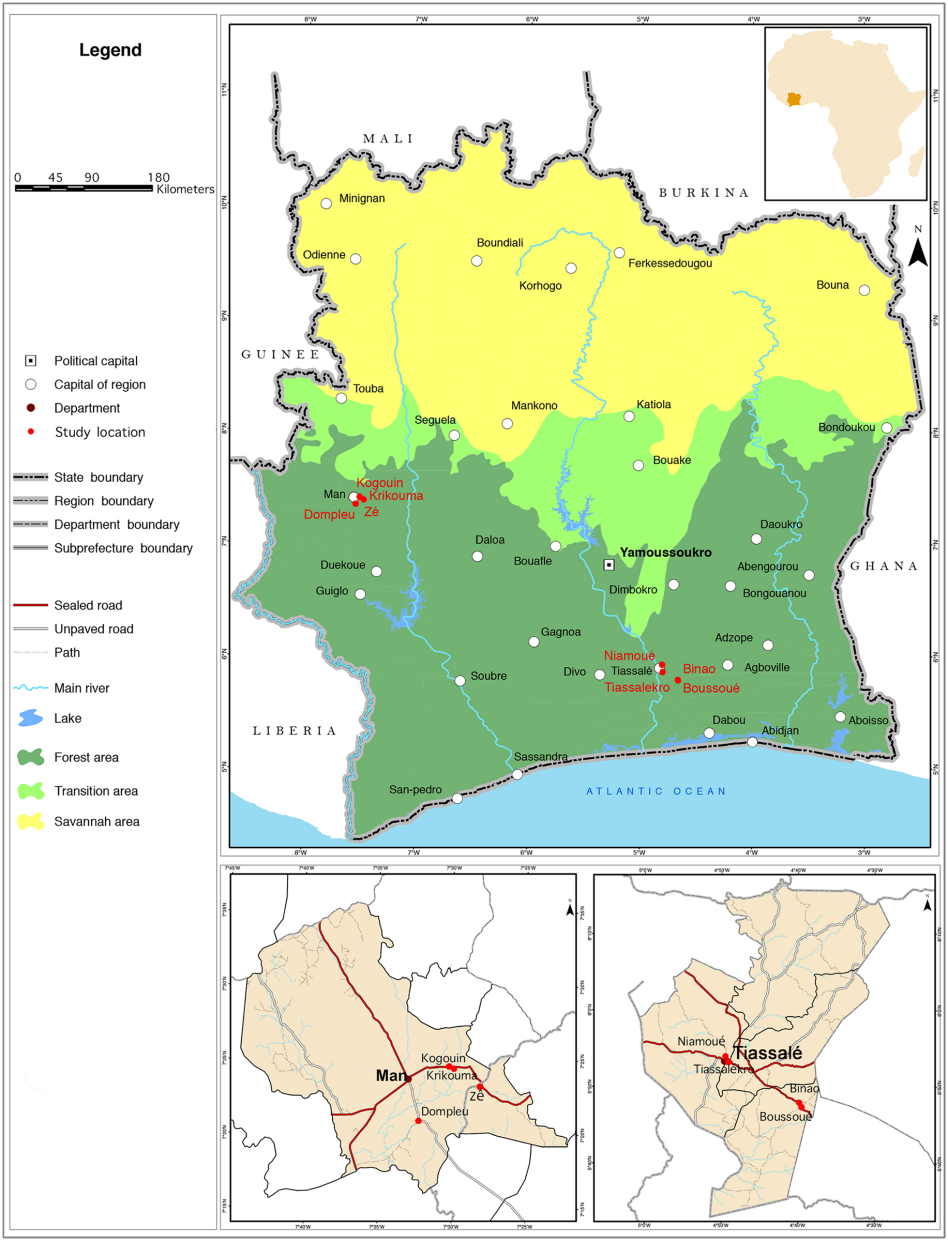
Map of the two study settings in Côte d’Ivoire.

The development of “*Koko et les lunettes magiques*” was done in three distinct phases (Fig 1). The first phase, carried out in four selected villages (two in south-central and two in western Côte d’Ivoire), consisted of preliminary research to assess KAPB of school-aged children (school enrolled and non-enrolled) regarding parasitic worm infections and hygiene, and to identify key messages to be included in the animated cartoon. Phase 2 entailed the cartoon production, which included the drafting of the script and story board and production of the initial version of the animated cartoon. The work was done in the Afrika Toon studios in Abidjan. In phase 3, we focused on school children, and hence, phase 3 took place in eight schools (four in south-central and four in western Côte d’Ivoire). Children from phase 1 were not necessarily part of phase 3. Based on specific feedback received from children, the cartoon was readily adapted and the final version produced. The choice of the two study settings for assessing KAPB and pilot-testing of the cartoon deliberately included different social-ecological contexts and was guided by previous research focusing on NTDs and diarrheal diseases [23–26]. Fig 2 displays the map with the two study settings.

The south-central part of Côte d’Ivoire is culturally very heterogeneous. In the Tiassalé region, there is an indigenous population (Elomoins, Abidjis, Agni, and Abbeys in the sub-prefecture of Tiassalé, and Souamlins, N’gbans, and Didas in the sub-prefecture of Taabo), a foreign-borne population (Baoulés, Attié, Senufo, Gouros, Yacouba, Malinké, and Abrons) and an allogenic population (originally from Burkina Faso, Niger, Nigeria, Ghana, Mali, Togo, and Benin). In the Tiassalé setting, phase 1 of the study took place in two villages, namely Niamoué and Tiassalékro. Phase 3 was carried out in four villages, namely Niamoué, Boussoué, Binao, and Tiassalékro. In this area, the villages have school groups (two schools or more in the same place) but we worked in only one school per village.

In western Côte d’Ivoire four main regions (Cavaly, Guemon, Haut-Sassandra, and Tonkpi), belonging to the district des Montagnes, are populated by four main ethnic groups, including Guéré, Toura, Wobé, and Yacouba. The ethnic composition in western Côte d’Ivoire is more homogeneous compared to the south-central part. Indeed, most people are native ethnic groups of the region. For this study we chose the Man setting in the Tonkpi region that has been the focus of our research activities since the late 1990s. Phase 1 of the study took place in Kogouin and Krikouma, while phase 3 took place in Dompleu, Zê, Krikouma, and Kogouin. Each village is small and has only one school.

### Three phases for the development of “*Koko et les lunettes magiques*”

#### Phase 1: Formative research

To identify key messages pertaining to parasitic worms, diarrhea, and hygiene-related practices that should be included in the cartoon, we carried out a KAPB study using the following methods: (i) questionnaire survey, (ii) focus group discussions (FGDs), and (iii) direct observations among children aged 9–14 years. The KAPB study was carried out in December 2013 and focused on knowledge regarding STHs (local terms, types of parasitic worms, transmission, and symptoms), treatment and prevention of STH infection, hygiene practices (after defecation and before eating), and information sources and preferred entertainment media (preferred information channel, favorite television show, and most appreciated cartoon). The target sample size was 120 children (80 school enrolled and 40 non-enrolled). The quantitative questionnaire utilized was pretested in a school that was not part of the presented study sites.

Eight FGDs were carried out with school children only, four each in the Tiassalé and Man regions. Each FGD included eight children (four girls and four boys). In direct observations it was assessed whether schools owned latrines (places of defecation), whether children were washing their hands after using the latrines, and whether they were using the bush instead of latrines. Questions or issues that were not addressed in the questionnaire were caught up in the interviews (FGDs) and observations. During the entire formative research, a cartoonist accompanied the social sciences team to become acquainted with the setting and obtain a deeper understanding of children’s risk behaviors.

#### Phase 2: Cartoon production

The whole process of cartoon production (including the changes made after the pilot testing) took place between December 2013 and August 2014, as summarized in Fig 1. First, an initial version of the script was written and the story board was produced by the cartoonists. Several iterations were made following discussions between the research team and the cartoonists so that key messages were well incorporated in the story and that the epidemiologic content was sound. Then the animated cartoon was drafted and corrections incorporated following further discussions with the research team. The first version of “*Koko et les lunettes magiques*” was completed in May 2014.

#### Phase 3: Pilot testing

In May 2014, following the production of the first complete version, each of the eight schools were visited and children invited to watch “*Koko et les lunettes magiques*”. Subsequently, children were administered a questionnaire in order to evaluate how well the cartoon messages resonated with them, whether or not children liked it, and perception of the overall length. The specific feedback from children and teachers guided the final fine-tuning of the cartoon production. Educators interested in participating watched the cartoon together with the school children and contributed qualitatively by making suggestions in separate interviews. Relevant observations were utilized for the improvement of the cartoon.

### Data analysis

The quantitative data collected from the KAPB questionnaires was double entered and cross-checked in EpiInfo version 6.04 (Centers for Disease Control and Prevention; Atlanta, GA, United States of America). Using STATA version 10 (Stata Corporation, College Station, TX, United States of America), frequency tables were generated. Qualitative data gathered from the FGDs were recorded, transcribed into Microsoft Word and then imported into MaxQDA version 1 (VERBI Software Consult; Berlin, Germany) for qualitative data analyses. The data were coded and analyzed to identify the frequency at which coded information and content categories occurred. A triangulation approach was used for analysis of the data derived from the three different methods (questionnaire, FGD, and observation).

## Results

### Phase 1: Formative research

#### Characteristics of the study participants

A total of 106 children, aged 9–14 years, were interviewed; 33 in Kogouin, 25 in Krikouma, 28 in Niamoué, and 20 in Tiassalékro. Eighty-two children (77%) attended school, while the remaining 24 children (23%) were not enrolled at school. Non-enrolled children in the selected communities were fewer than expected and the planned number of 40 non-enrolled children could not be reached.

#### Knowledge regarding STHs

Children’s knowledge of parasitic worms was investigated regarding causes (transmission), symptoms, and available treatment. In the FGDs, children reported that “*worms give diseases*”, “*worms kill people*”, “*worms give belly aches*”, and “*they consume your blood and eat your vitamin*”. However, children were unable to attribute scientific names/medical terms to parasitic worms and commonly used words such as microbes, worms, Guinea worms, and leeches. According to the children, there are several types of worms (e.g., small, long, large, round, or thin) and they may have different colors (e.g., white, red, black, or brown).

Table 1 shows the results obtained from the questionnaire survey. Overall, 68% of the children knew about the existence of parasitic worms with no significant difference between children enrolled and non-enrolled in school. The main causes of parasitic worms reported were the consumption of sweet food, eating without washing hands, sitting on the floor, and consumption of spoiled food. A considerable proportion of children did not know how parasitic worms are transmitted, with little difference among enrolled and non-enrolled children. For example, 17% of non-enrolled children thought that sweet food causes worm infections, compared to 11% of enrolled children. Importantly though, non-enrolled children never mentioned the lack of hand washing as a source of infection of parasitic worms, while walking without shoes was mentioned by 13% of the non-enrolled children, but none of the enrolled children. Further results, stratified by enrolled and non-enrolled children, are presented in S1 Table.

**Table 1:** Results pertaining to knowledge on parasitic worms among school-aged children in south-central (Tiassalé) and western (Man) Côte d’Ivoire during phase 1.

Variables	Tiassalé		Man	
	Niamoué (%)	Tiassalékro (%)	Kogouin (%)	Krikouma (%)
**Causes (transmission) of prasitic worms**	**N = 25**	**N = 17**	**N = 30**	**N = 21**
Spoiled food	3 (12)	3 (18)	2 (7)	4 (19)
Eat without washing hands	3 (12)	6 (35)	8 (27)	0 (0)
Dirty water	0 (0)	0 (0)	0 (0)	2 (10)
Play in dirty water	0 (0)	1 (6)	0 (0)	1 (5)
Sit on the ground	0 (0)	1 (6)	0 (0)	0 (0)
Water	0 (0)	1 (6)	0 (0)	0 (0)
Garbage	1 (4)	2 (12)	3 (10)	1 (5)
Walk without shoes	0 (0)	0 (0)	1 (3)	2 (10)
Not purge oneself	1 (4)	0 (0)	0 (0)	0 (0)
Sweet food	8 (32)	1 (6)	9 (30)	3 (14)
Don’t know	7 (28)	0 (0)	7 (23)	7 (33)
Other	2 (8)	2 (12)	0 (0)	1 (5)
**Symptoms of parasitic worms**	**N = 28**	**N = 20**	**N = 33**	**N = 25**
General fatigue	17 (61)	6 (30)	21 (64)	17 (68)
Blindness	3 (11)	1 (5)	4 (12)	2 (8)
Constipation	2 (71)	2 (10)	11 (33)	2 (8)
Diarrhea	17 (61)	16 (80)	21 (64)	19 (76)
Overweight	1 (4)	2 (10)	3 (9)	2 (8)
Stunting	11 (39)	15 (75)	19 (58)	17 (68)
Lack of concentration	10 (36)	11 (55)	17 (52)	11 (44)
Lack of appetite	16 (57)	14 (70)	24 (73)	14 (56)
Stomach aches	19 (68)	17 (85)	23 (70)	19 (16)
Cough	9 (32)	2 (10)	14 (42)	9 (36)
Don’t know	0 (0)	0 (0)	1 (3)	1 (4)
Other	6 (21)	0 (0)	2 (6)	3 (12)
**Place of treatment**	**N = 24**	**N = 17**	**N = 30**	**N = 20**
Hospital	22(92)	17 (100)	23 (77)	16 (80)
Pharmacy	1 (4)	0 (0)	2 (7)	0 (0)
Traditional medicine	0 (0)	0 (0)	3 (10)	0 (0)
Don’t know	0 (0)	0 (0)	2 (7)	1 (5)
Other	1 (4)	0 (0)	0 (0)	3 (15)

The questionnaire findings were confirmed by the FGDs. Children said: *“When you eat too much sweet banana*, *it causes worms in the belly”* and *“You have worms when you always eat sweet foods”*. In addition, the concept of dirt appears in conjunction with consumption of food and water: “*We must not collect things from the ground to eat”* or *“One should not drink any kind of water because there is water that if you drink it*, *you’ll have worms”*. Walking and playing on dirty ground was also mentioned as a source of contamination: “*If you go to places where people throw garbage*, *you’ll have worms*” or “*If you walk on worms*, *they will come into your feet and after enter into your belly*”. Moreover, children mentioned that playing in dirty places and eating without washing hands were causes of parasitic worm infections.

In terms of symptoms related to parasitic worms that may give rise to recognize parasitic worm diseases, Table 1 shows that children put forth abdominal pain, diarrhea, and lack of appetite. Failure to grow appeared to be an important symptom of parasitic worm infections for children in Tiassalékro, whereas general fatigue was mentioned by children in Niamoué, Krikouma, and Koguoin. During the FGDs, additional symptoms were identified such as vertigo, nausea, and hunger, encapsulated by the following statement: “*When a person is infected with worms*, *he will be hungry just after eating”*.

Most of the children knew that they should go to a health care center for treatment if they experienced symptoms of worm diseases. In Kogouin, 10% of the children reported that parasitic worms can also be treated with traditional medicine (Table 1). Some children reported that parents went directly to a pharmacy to obtain treatment. The FGDs revealed that treatment for parasitic worm infections mainly occurs at home or at school: “*When you have worms in your belly*, *they give you drugs at school which kill them*”. The exact names of drugs used to kill parasitic worms were not known by children.

#### Attitudes and practices

Table 2 shows that, particularly in Niamoué and in Krikouma, children thought that they could contract parasitic worms through specific behaviors, such as sweet fruit consumption, drinking unsafe water, eating dirty and spoiled food, and playing without wearing shoes. Children were thus afraid of becoming infected with worms. FGDs highlighted that this concern is due to the perception that worms in the stomach “*can cause other diseases such as diarrhea*, *measles*, *fever*, *typhoid*, *or dysentery*”. For some of the children, parasitic worms can lead to death: “*I’m afraid because I can die*.” For others, the concern was due to the doubt caused by the presence of parasitic worms in the stomach: “*We don’t know what it can cause in your belly*” and for those who knew the consequences of parasitic worms it is “*because when you eat*, *your belly will hurt*”, “*worms in your belly will drink your blood*”, and “*they also remove the vitamin in your body*”. Finally, parasitic worms are attached with economic costs because parents will bring the child to the hospital and spend money. In contrast, children in Tiassalékro (60%) and Kogouin (55%) believed they cannot contract intestinal worms because they do not play near the garbage, they wash hands before eating, they eat well, and they can be dewormed. For these reasons, they were not afraid of having worms (93% in Tiassalékro and 90% in Koguoin).

**Table 2:** Attitudes pertaining to knowledge on parasitic worms among school-aged children in south-central (Tiassalé) and western (Man) Côte d’Ivoire during phase 1.

Variables	Tiassalé		Man	
	Niamoué (%)	Tiassalékro (%)	Kogouin (%)	Krikouma (%)
**Possibility of contracting worms**				
Yes	19 (68)	8 (40)	15 (46)	13 (52)
No	9 (31)	12 (60)	18 (55)	12 (48)
**Afraid of having worms**				
Yes	16 (64)	14 (93)	26 (90)	18 (86)
No	9 (36)	1 (7)	3 (10)	3 (14)

With regard to practices, Table 3 shows that children stated to always wear shoes when going to the field with their parents. Most children reported to wear shoes at school, but at home, the proportion of children wearing shoes was lower. About 80% of children declared to defecate in latrines at home, in all study villages. However, some children said they additionally defecated at the shores of the river. More than half of the children reported to wash their hands with water and soap after defecation. About a third of the children interviewed never washed hands with water and soap before eating. Most children reported to wash fruits before eating them. Children enrolled in school reported significantly more often to wash their hands with water and soap after defecation compared to non-enrolled children (50% *vs*. 8%). A similar pattern was found for hand washing before food consumption (Table 3).

**Table 3:** Practices pertaining to parasitic worms among school aged children in south-central (Tiassalé) and western (Man) Côte d’Ivoire during phase 1.

Variables	Tiassalé		Man	
	Niamoué (%)	Tiassalékro (%)	Kogouin (%)	Krikouma (%)
**Wearing shoes in field**				
Never	0 (0)	0 (0)	1 (6)	0 (0)
Sometimes	3 (19)	2 (22)	2 (11)	2 (9)
Always	13 (81)	7 (78)	15 (83)	21 (91)
**Wearing shoes at school**				
Never	0 (0)	0 (0)	0 (0)	0 (0)
Sometimes	0 (0)	2 (11)	3 (13)	1 (6)
Always	18 (100)	16 (89)	20 (87)	17 (94)
**Wearing shoes at home**				
Never	0 (0)	1 (6)	3 (9)	1(4)
Sometimes	13 (46)	5 (28)	16 (49)	9 (36)
Always	15 (54)	12 (67)	14 (42)	15 (60)
**Defecation area of family members**				
Field	1 (4)	1 (5)	3 (9)	4 (16)
Latrine at home	22 (79)	15 (75)	26 (79)	21 (84)
Latrine at school	0 (0)	1 (5)	0 (0)	0 (0)
River	0 (0)	1 (5)	0 (0)	0 (0)
Other	5 (18)	2 (10)	4 (12)	0 (0)
**Children’s defecation areas**				
Field	3 (11)	1 (5)	3 (9)	4 (17)
Latrine at home	19 (68)	17 (85)	25 (76)	19 (83)
Latrine at school	0 (0)	1 (5)	0 (0)	0 (0)
River	6 (21)	1 (5)	5 (15)	2 (9)
**Hand washing with water and soap after defecation**				
Never	5 (19)	1 (5)	5 (16)	6 (25)
Sometimes	7 (27)	4 (20)	6 (19)	3 (13)
Always	14 (54)	15 (75)	21 (66)	15 (63)
**Hand washing with water and soap before eating**				
Never	10 (36)	7 (35)	7 (21)	11 (44)
Sometimes	7 (25)	4 (20)	7 (21)	1 (4)
Always	11 (39)	9 (45.0)	19 (58)	13 (52)
**Wash fruit before eating**				
Never	8 (29)	6 (32)	10 (30)	6 (24)
Sometimes	7 (25)	4 (21)	4 (12)	4 (16)
Always	13 (46)	9 (47)	19 (58)	15 (60)

#### Source of information

Table 4 summarizes results pertaining to sources of information regarding health education, specifically from where information was received and what kind of information tools children like. In the four villages, children reported to have already received information on parasitic worms in the past at school through the teachers and at home through family members. During FGDs, the griot (a West African historian, storyteller, poet, or musician who can simply also spread information in a village) was mentioned as additional source is the village: “*I also heard that there are people who use a microphone to talk and say that after playing*, *if your hands are dirty*, *you must wash them before eating to avoid worms in your belly*”. Children’s preferred sources of information are books and television. Some children also wished to be informed by the members of their families (“*through the advice of our parents…”*) and in the health centers (“*I want the doctors to teach us how worms enter in our belly*, *develop themselves in our body and give us diseases*”).

**Table 4:** Sources of information received by children and additional information sources desired about parasitic worms and diarrhea from KABP studies in four villages of south-central (Tiassalé) and western (Man) Côte d’Ivoire during phase 1.

Variable	Tiassalé		Man	
	Niamoué (%)	Tiassalékro (%)	Kogouin (%)	Krikouma (%)
**Sources of received information**				
By the teacher	8 (33)	5 (19)	9 (38)	8 (42)
Reading	5 (21)	7 (26)	5 (21)	2 (11)
Film	2 (8)	6 (22)	4 (17)	3 (16)
School exercises	1 (4)	2 (7)	0 (0)	1 (5)
In the family	8 (33)	7 (26)	6 (25)	5 (26)
**Desired sources of information**				
Reading	15 (65)	10 (56)	16 (50)	12 (48)
Comic reading	0 (0)	2 (11)	0 (0)	0 (0)
Television	8 (35)	6 (33)	16 (50)	13 (52)

### Phase 2: Production of animated cartoon

**Fig 3:**
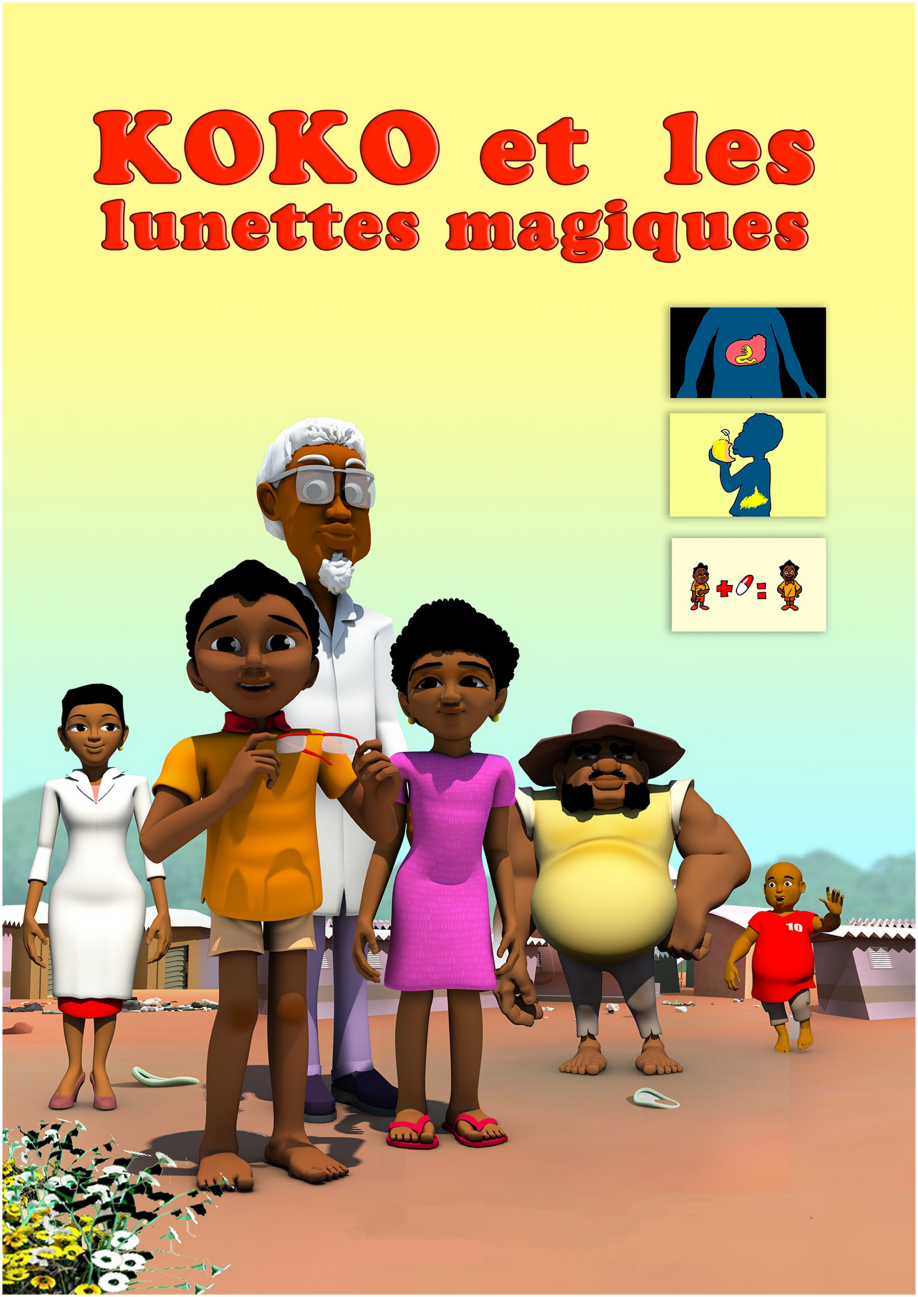
Poster “*Koko et les lunettes magiques*”.

**Fig 4:**
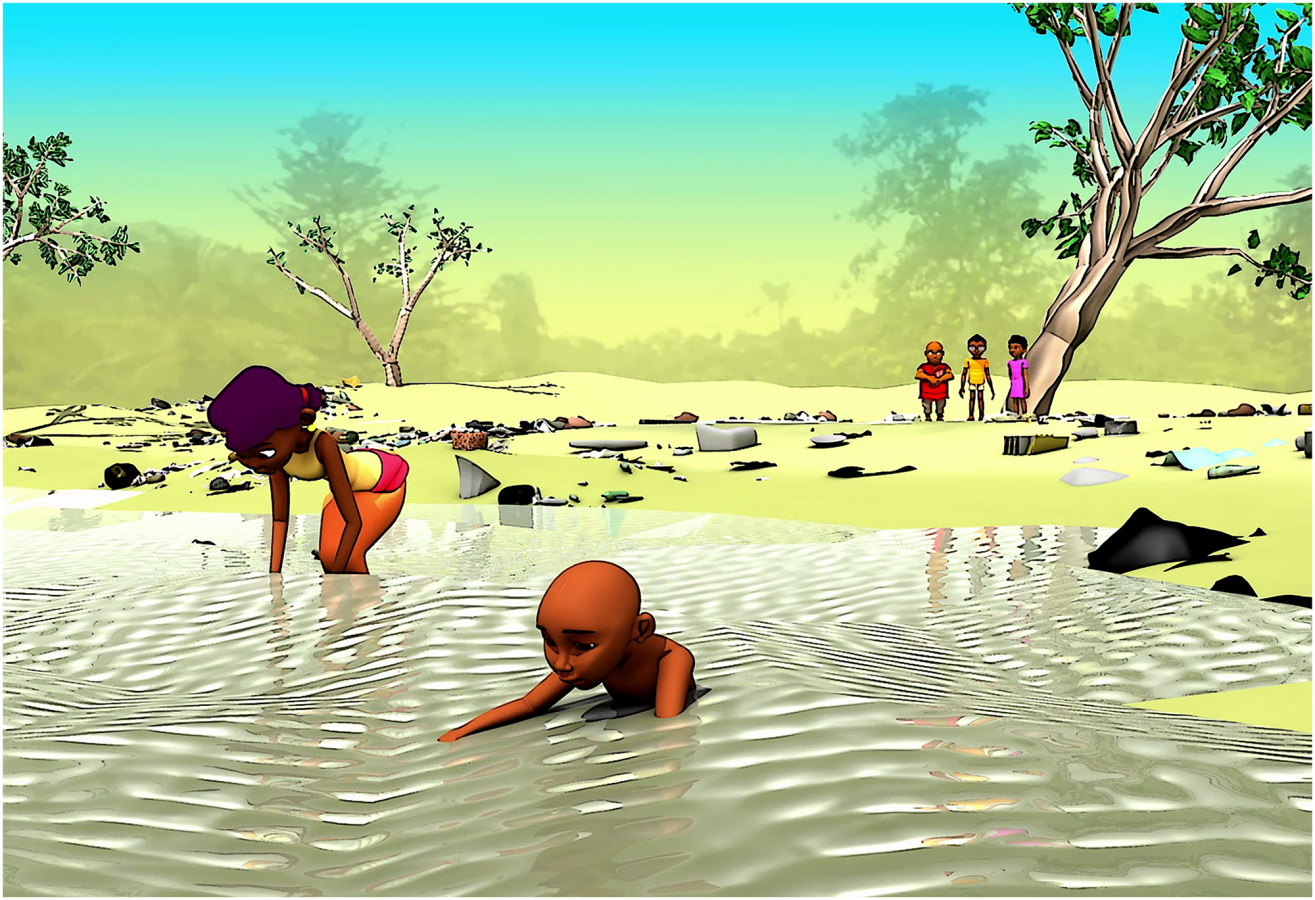
Extract from the animated cartoon “*Koko et les lunettes magiques*”. The 3D image shows Koko and his friends observing risk behavior of community members.

**Fig 5:**
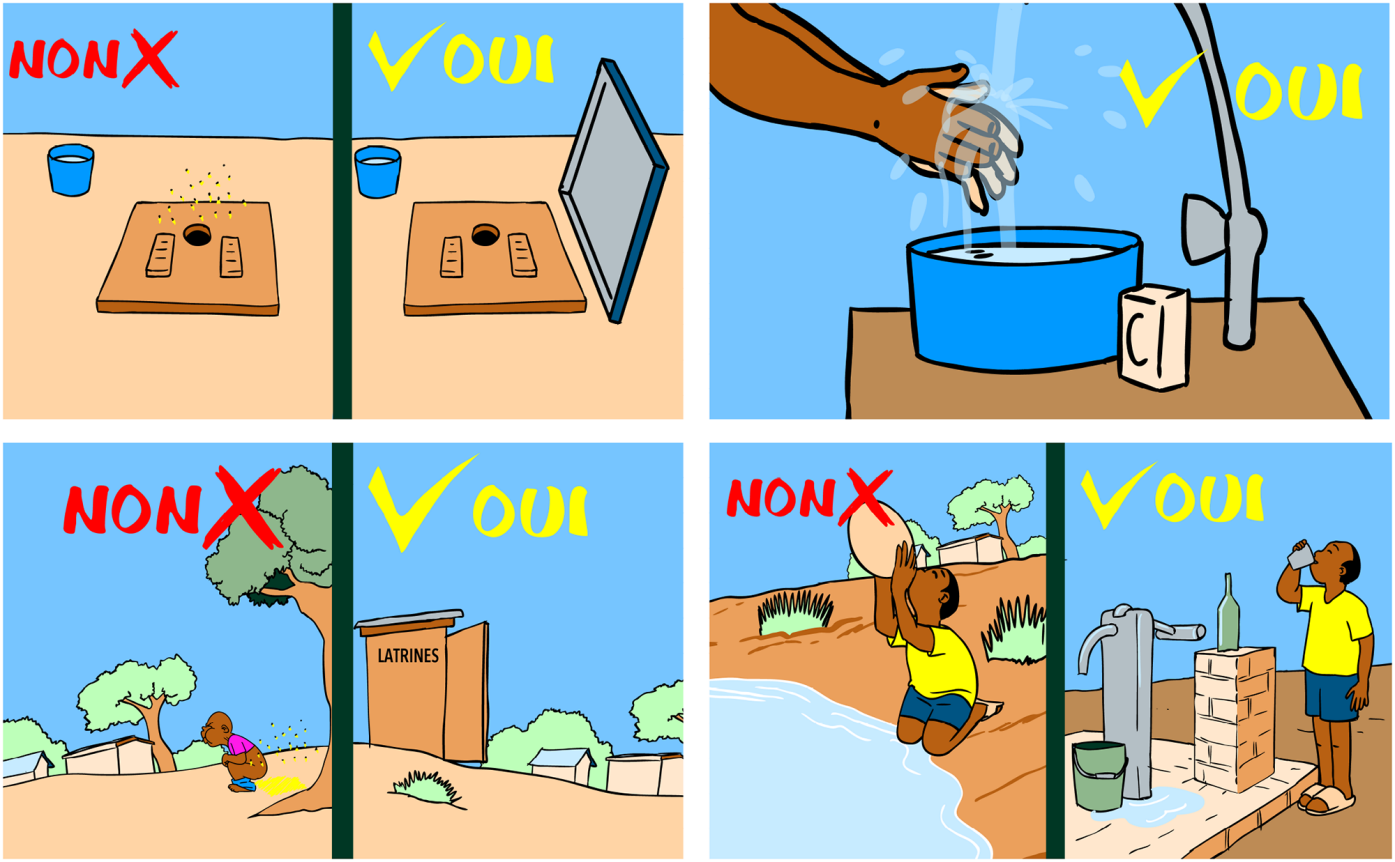
Extracts from the animated cartoon “*Koko et les lunettes magiques*”. The images show the final part of the animated cartoon where key messages are reinforced. Images of this last section are shown in 2D.

The animated cartoon was produced by Afrika Toon, in a transdisciplinary collaboration with the scientific team of the project (epidemiologists, civil engineers, and social scientists) and the communities (school children and teachers; see pilot-testing below). In brief, “*Koko et les lunettes magiques*” is a 15-minute story about clean water, use of latrines, and hand washing with the aim of improving health- and hygiene-related knowledge and practices towards preventing intestinal worm infections and improving hygiene (see Fig 3 displaying the poster of the animated cartoon). Koko is an 8-year-old boy living in the village Popokro, who is confronted with intestinal worm infections and inappropriate hygiene behavior. Koko himself suffers from intestinal worms. The village doctor provides him magic glasses (*lunettes magiques*; in French) that allow Koko to see the environment as if he was seeing through a microscope. Hence, Koko sees the parasites and how the transmission to humans occurs (Fig 4). Koko and his friends then decide that they need to take action and inform the community about the dangers related to these infections and come forward with preventive strategies. Toward the end of the animated cartoon, the most important key messages are re-iterated, comparing appropriate and inappropriate behaviors (Fig 5).

Box 1 displays the key messages that were incorporated into the story, following the KAPB results from the preliminary assessment. Particular emphasis was put into using a language adapted to school-aged children. Thus, simple and known terms (e.g., worms, microbes, and parasites) identified during the KAPB assessment, were used instead of less accessible medical terms. The final version of “*Koko et les lunettes magiques*” is available online (https://www.youtube.com/watch?v=PCNLEK5Ityw).

**Box 1. Key messages.**

List of the key messages formulated by the research team following the preliminary assessment. It includes messages that aim to increase the knowledge on good hygiene practices, while filling identified knowledge gaps and re-enforcing already existing knowledge.

- Always use latrines and avoid open defecation (so that everyone can live in a healthy environment)
- Always wash your hands with water and soap after defecation (because otherwise you can contaminate food and other objects/people with your hands)
- Always wash your hands with water and soap before eating (to avoid ingesting microbes that will make you sick)
- Always wash fruits and vegetables with water before eating them raw (to avoid ingesting microbes that will make you sick)
- Do not walk and play outdoors without shoes to avoid parasitic worm infections
- You must go to the hospital when you have diarrhea and abdominal pain
- Always cover the latrine to prevent the spread of flies (they can contaminate the food and make you sick)
- Fruits well washed and consumed in moderation are healthy and do not cause diarrhea
- You must drink clean water (clean water source, treated water) to prevent diarrheal diseases (because dirty water harbors germs that will make you sick)
- Always cover food to avoid flies on it, as they can contaminate food with germs that will make you sick
- Avoid bathing in the river, stream, lake or creek because there is a risk of getting infected with schistosomiasis
- When a child has intestinal worms, its physical and cognitive development may be impaired and it cannot concentrate at school

### Phase 3: Pilot testing

The pilot testing was conducted in eight schools. Four were in the Tiassalé region (Binao, Boussoué, Niamoué, and Tiassalékro) and four in the Man region (Krikouma, Kogouin, Zê, and Dompleu). All children from grades 3–5 were invited to watch the cartoon. A total of 401 children participated in the KAPB survey.

#### Appreciation of “*Koko et les lunettes magiques*” by school children

Most children who watched the cartoon reported to like it (Tiassalé region: 82.0% and Man region: 85.1%). Table 5 shows that, in all eight villages, the cartoon was not perceived to be too long, but rather too short, particularly for children in the Tiassalé (55.0%) region. The cartoon was considered to be very funny both in Tiassalé (79.0%) and in Man (79.0%). Most of the children found the cartoon interesting and they could readily grasp the key messages. Yet, both children and teachers pointed out some specific terms and sequences of the story that were difficult to understand. Thus, the language was further simplified and relevant sequences revisited.

**Table 5:** Appreciation of “*Koko et les lunettes magiques*” by children in south-central (Tiassalé) and western (Man) Côte d’Ivoire during phase 3. A questionnaire was administered to 401 children of eight schools (four in Tiassalé and four in Man) after screening the animated cartoon.

Variable	Tiassalé (%)	Man (%)	Total (%)
**Too long**			
Yes	25 (12.5)	40 (19.9)	65 (16.2)
No	175 (87.5)	161 (80.1)	336 (83.8)
**Too short**			
Yes	110 (55.0)	28 (13.9)	138 (34.4)
No	90 (45.0)	173 (86.1)	263 (65.6)
**Funny**			
Yes	158 (79.0)	157 (78.1)	315 (78.6)
No	42 (21.0)	44 (21.9)	86 (21.4)
**Boring/wearisome**			
Yes	8 (4.0)	11 (5.5)	19 (4.7)
No	192 (96.0)	190 (94.5)	382 (95.3)
**Complicated/difficult**			
Yes	42 (21.0)	81 (40.3)	123 (30.7)
No	158 (79.0)	120 (59. 7)	278 (69.3)

#### Knowledge about STHs after screening of the animated cartoon

The questions posed to children pertained only to specific information that was provided through the animated cartoon. After screening, most children in Tiassalé and in Man knew that people should defecate in latrines. Only a few said that they could also defecate in the bush. Hand washing after defecation was recognized as important hygiene behavior (97.0% in Tiassalé, 98.5% in Man). In addition, hand washing before eating was considered a good practice. Finally, children were aware that fruits should be washed before eating (98.5% in Tiassalé and 87.0% in Man).

Children reported that people should consume water obtained from pumps rather than from unprotected surface. For some children in Boussoué (16.0%), Niamoué (34.0%), and Zê (14.0%), however, villagers could also consume creek and river water. Furthermore, children reported that food should be covered to prevent contamination. Only a small proportion of children referred to flies as a means of transmitting pathogens. Wearing shoes was recommended by children because walking barefoot can result in worm infections. With regard to the reasons given for not bathing in the river, children in Man cited more schistosomiasis compared to those from Tiassalé who mentioned dirt in the river and creek. These results are presented in more detail in S2 Table.

Stomach aches were the most frequently reported sign of parasitic worms in all villages surveyed, while diarrhea was also frequently mentioned. In Tiassalé, only diarrhea and stomach ache were mentioned as suggestive signs for the presence of parasitic worms. In Man, additional symptoms were given, such as general fatigue, lack of concentration at school, failure to thrive, lack of appetite, and incapacity to think clearly (see S3 Table for more details).

Children considered health centers as the primary location where people with parasitic worms should seek treatment. In Kogouin and Zê, children also mentioned that people could be treated in the pharmacy, after they bought the deworming drugs.

## Discussion

STH infections, schistosomiasis, and diarrhea are among the most common infectious diseases in LMICs and they cause a considerable global burden [27]. WHO recommends preventive chemotherapy as the key strategy for morbidity control due to STHs and schistosomiasis [7,28]. Indeed, in 2015, an estimated 566.7 million doses of albendazole or mebendazole have been administered to preschool- and school-aged children requiring preventive chemotherapy against STHs and 65.2 million doses of praziquantel have been administered against schistosomiasis [29]. Whenever resources allow, integrated control approaches should be implemented that include access to clean water and improved sanitation, along with health education to change behavior [30,31]. It is conceivable that such integrated control approaches have a considerable impact on diarrheal diseases as well. Building upon the experience from previous work on educational videos that have proven to be effective at improving children’s knowledge and changing their attitudes and behaviors [22,32–34], our aim was to develop a health education tool to improve school children’s awareness regarding STH, schistosomiasis, diarrheal diseases, and hygiene-related practices.

WHO defines health education as follows: “Health education is a process comprising of consciously constructed opportunities for learning and communication designed to improve health information, health literacy, health knowledge and developing life skills which are conducive to the promotion of an individual and community’s health including that of the environment” [35]. The aim of health education is to change human behavior by increasing awareness of the health and social impacts of a disease [17,36]. Of note, the value of moving images in health education has been highlighted by WHO as early as 1988 [37]. When designing a health education tool or intervention, it is essential to understand the baseline knowledge of the target population and to tailor the messaging to local contexts and needs. For characterizing the baseline situation, KAPB studies are a promising approach, using different tools such as questionnaires, FGDs, and direct observations, as done in the current study and elsewhere in Côte d’Ivoire and abroad [13,23]. Findings from KAPB studies allow the identification of appropriate and culturally adapted key messages for integration into health education tools. Thus, the tools can take into account a particular context and the socio-cultural organization of the target population, focusing on precise purposes, e.g., filling identified knowledge gaps in a way that is contextually appropriate and culturally acceptable. For example, although Koko is a school-aged child, we opted to tell a story in the community rather than in a school because a considerable proportion of school-aged children still do not have access to primary school education. Furthermore, still today, the opinions of the family heads and the community elderly are very important in rural Côte d’Ivoire as in many other West African settings. It becomes apparent that telling a story adapted to the local socio-cultural context can maximize the impact of an intervention and is more likely to make change happen. The preliminary assessment allowed us to identify some misconceptions in the school-aged population (e.g., eating sweet fruit as cause of worm disease and diarrhea). Thus, we could address this issue in a key message of the animated cartoon, by emphasizing that consumption of fruit is healthy, although they need to be washed beforehand.

We observed specific differences in the knowledge of children regarding the transmission of parasitic worm infections. Indeed, in some communities, the majority of children believed that they were at risk and were afraid of contracting parasitic worms because of their behaviors (i.e., sweet fruit consumption, drinking unsafe water, eating dirty and spoiled food, and playing outdoors without wearing shoes). In contrast, in other communities, a large proportion of children were not afraid because they reported to not put themselves at risk and if they did contract parasitic worms, it was possible to remedy this situation through access to deworming drugs. The latter part of children were those living in villages close to the towns of Man and Tiassalé, thus we conjecture that they had better access to information and treatment and thus were less afraid of parasitic worm diseases. This emphasizes the importance of assessing the KAPB in a representative sample of the target population before developing a health educational tool.

“*Koko et les lunettes magiques*” targets a very specific population, namely school-aged children. Indeed, the main character of the animated cartoon is an 8-year-old boy and the story is designed in such a way that the audience (children) can identify themselves with Koko. Nonetheless, other adult characters (Koko’s father, a nurse, a wise doctor, an old man, and Koko’s friends) are also integrated in the story. Hence, adults can also identify themselves with the story told. The animated cartoon was developed for school-aged children and discussions are underway with the education sector how to integrate it into the school curriculum. However, the cartoon can also be screened at public places for entertaining and educating adults, since it simplifies complex issues.

“*Koko et les lunettes magiques*” can be seen as an entry point of a larger health education intervention package that will allow rapidly attracting children’s (and adults’) attention. However, it is essential that messages are reiterated during discussions, drawing assignments or group work (the type and combination of these methods would depend whether the intervention is school-based or community-based) in order to consolidate the knowledge gained and to make sure that the most important messages are understood correctly. Indeed, the pilot testing revealed that although children retain most of the key messages right after the screening [38], some knowledge gaps still remain. Furthermore, repetition of key messages should ideally be done not only right after the screening but also over time, so that messages can be retained over the longer term. Moreover, it could be of great value to involve teachers by increasing their urge to make sure their schools have available clean water for drinking and hand washing facilities as well as soap and properly maintained hygienic facilities for defecation. Teachers can also be trained to regularly update their students’ knowledge regarding hygiene- and health-related practices. Such interventions could be supplemented by participation of parents and the community, thus encouraging them to take an active role in the health education of their children [13]. When the aforementioned issues are considered, the decision to adopt a good health practice, however, is also matter of individual choice. The health believe model presented by Rosenstock in 1974 assumes that people will only act to prevent a disease if they have minimal knowledge of health, whereas health needs to be an important dimension in their life. The perception of the threat to health and belief in the effectiveness of the action to reduce this threat will determine a preventive action [39–40].

In conclusion, “*Koko et les lunettes magiques*” was developed to educate school-aged children in an entertaining and context-specific manner. Clearly, children liked the animated cartoon and the pilot testing revealed that children could keep the transmitted information shortly after they were shown the cartoon. In a recent intervention study in 25 schools of western Côte d’Ivoire the cartoon has been tested for its effect on KAPB in the short term and results of the study will be presented elsewhere. An important next step will be to validate the cartoon on its effect on the KAPB regarding STH infections, schistosomiasis, and diarrheal diseases in the mid- and long-term. It is essential to validate such health education tools in different social-ecological contexts in order to create evidence-based and locally adapted designs for sustainable integration of health educational packages into school curricula along with regional deworming programs against STHs, schistosomiasis, and other NTDs.

## Supporting information

S1 Table Differences between enrolled and non-enrolled children regarding STH knowledge during phase 1 in four villages of south-central (Tiassalé) and western (Man) Côte d’Ivoire. (DOCX)

S2 Table Hygiene measures reported by school children following the screening of “*Koko et les lunettes magiques*” during the pilot testing in eight schools of south-central (Tiassalé) and western (Man) Côte d’Ivoire. (DOCX)

S3 Table Knowledge of symptoms caused by worms following the screening of “*Koko et les lunettes magiques*” during the pilot testing in eight schools of south-central (Tiassalé) and western (Man) Côte d’Ivoire. (DOCX)
