# Supplementary material for: “Koko et les lunettes magiques”: An educational entertainment tool to prevent parasitic worms and diarrheal diseases in Côte d’Ivoire
Source: PLoS Negl Trop Dis. 2017 Sep 21;11(9):e0005839. doi: 10.1371/journal.pntd.0005839 (PMC5630154; doi:10.1371/journal.pntd.0005839)
Supplement: S1 Table — (DOCX) [file pntd.0005839.s001.docx]

**S1 Table.** Differences between enrolled and non-enrolled children regarding STH knowledge during the Phase 1 in four villages of south central (Tiassalé) and western (Man) Côte d’Ivoire.

| **Variables Non- enrolled (%) Enrolled (%) Total** | | | | | | |
| --- | --- | --- | --- | --- | --- | --- |
| **Causes (transmission) of intestinal worms** | **N=23** |  |  | **N=70** |  | **N=93** |
| Spoiled food | 4 (17.4) |  |  | 8 (11.4) |  | 12 (12.9) |
| Eat without washing hands | 0 (0.0) |  |  | 17 (24.3) |  | 17 (18.3) |
| Drink dirty water | 0 (0.0) |  |  | 2 (2.9) |  | 2 (2.2) |
| Play in dirty water | 0 (0.0) |  |  | 2 (2.9) |  | 2 (2.2) |
| Sit on the ground | 0 (0.0) |  |  | 1 (1.4) |  | 1 (1.1) |
| Water | 0 (0.0) |  |  | 1 (1.4) |  | 1 (1.1) |
| Garbage | 0 (0.0) |  |  | 7 (10.0) |  | 7(7.5) |
| Walk without shoes | 3 (13.0) |  |  | 0 (0.0) |  | 3 (3.2) |
| Not purge oneself | 1 (4.3) |  |  | 0 (0.0) |  | 1 (1.1) |
| Sweet food | 4 (17.4) |  |  | 17 (24.3) |  | 21 (22.6) |
| Don’t know | 10 (43.4) |  |  | 11 (15.7) |  | 21 (22.6) |
| Other | 1 (4.3) |  |  | 4 (5.7) |  | 5 (5.4) |
| **Symptoms of intestinal worms** | **N=24** |  |  | **N=82** |  | **N=106** |
| Fatigue | 12 (50.0) |  |  | 49 (59.8) |  | 61 (55.6) |
| Blindness | 1 (4.2) |  |  | 9 (11.0) |  | 10 (9.4) |
| Constipation | 6 (25.0) |  |  | 14 (17.1) |  | 17 (16.0) |
| Diarrhea | 13 (54.2) |  |  | 60 (73.2) |  | 73 (68.9) |
| Over weight | 2 (8.3) |  |  | 6 (7.3) |  | 8 (7.6) |
| Stunting | 9 (37.5) |  |  | 53 (64.6) |  | 62 (58.5) |
| Lack of concentration | 0 (0.0) |  |  | 49 (59.8) |  | 49 (46.2) |
| Lack of appetite | 14 (58.3) |  |  | 54 (65.9) |  | 68 (64.2) |
| Stomach aches | 17 (70.8) |  |  | 61 (74.4) |  | 78 (73.6) |
| Cough | 4 (16.7) |  |  | 30 (36.6) |  | 34 (32.1) |
| Don’t know | 1 (4.2) |  |  | 1 (1.2) |  | 2 (1.9) |
| Other | 6 (25.0) |  |  | 5 (6.1) |  | 11 (10.4) |
| **Place of treatment** | **N=24** |  |  | **N=67** |  | **N=91** |
| Hospital | 16 (66.6) |  |  | 62 (92.5) |  | 78 (87.6) |
| Pharmacy | 1 (4.1) |  |  | 2 (3.0) |  | 4 (4.5) |
| Traditional medicine | 3 (12.5) |  |  | 0 (0.0) |  | 3 (3.4) |
| Don’t know | 2 (8.3) |  |  | 1 (1.5) |  | 3 (3.4) |
| Other | 2 (8.3) |  |  | 2 (3.0) |  | 3 (3.4) |
