# Supplementary material for: “Koko et les lunettes magiques”: An educational entertainment tool to prevent parasitic worms and diarrheal diseases in Côte d’Ivoire
Source: PLoS Negl Trop Dis. 2017 Sep 21;11(9):e0005839. doi: 10.1371/journal.pntd.0005839 (PMC5630154; doi:10.1371/journal.pntd.0005839)
Supplement: S2 Table — (DOCX) [file pntd.0005839.s002.docx]

**S2 Table.** Hygiene measures reported by schoolchildren following the screening of “Koko et les lunettes magiques” during the pilot testing in eight schools of south-central (Tiassalé) and western (Man) Côte d’Ivoire.

| **Variable** | **Tiassalé** |  |  |  |  | **Man** |  |  |  |
| --- | --- | --- | --- | --- | --- | --- | --- | --- | --- |
|  | **Binao**  **(%)** | **Boussoué**  **(%)** | **Niamoué**  **(%)** | **Tiassalékro (%)** |  | **Dompleu**  **(%)** | **Kogouin**  **(%)** | **Krikouma**  **(%)** | **Zê**  **(%)** |
| **Defecation area** |  |  |  |  |  |  |  |  |  |
| Latrine | 50(100.0) | 49(98.0) | 48(96.0) | 50(100.0) |  | 49(96.1) | 49(98.0) | 50(100.0) | 48(96.0) |
| Field | - | 1 (2.0) | 1 (2.0) | - |  | 2 (3.9) | 1 (2.0) | - | - |
| River | - | - | 1 (2.0) | - |  | - | - | - | - |
| Other | - | - |  | - |  | - | - | - | - |
| **Hand Washing after defecation** |  |  |  |  |  |  |  |  |  |
| Nothing | - | 1 (2.0) | - | - |  | - | - | - | 1 (2.0) |
| Wash with water | 2 (4.0) | 2 (4.0) | 3 (6.0) | 3 (6.0) |  | 13 (25.5) | 3 (6.0) | 1 (2.0) | 5 (10.0) |
| Wash with water and soap | 48 (96.0) | 47 (94.0) | 47 (94.0) | 47(94.0) |  | 36(70.6) | 47(94.0) | 49(98.0) | 42 (84.0) |
| Other | - | - | - | - |  | 2 (3.9) | - | - | 2 (4.0) |
| **Hand washing before eating** |  |  |  |  |  |  |  |  |  |
| Nothing | **-** | **-** | **-** | **-** |  | **-** | **-** | **-** | **-** |
| Wash with water | 2 (4.0) | 2 (4.0) | 2 (4.0) | - |  | 3 (5.8) | 2 (4.0) | 4 (8.0) | 1 (2.0) |
| Wash with water and soap | 48 (96.0) | 48 (96.0) | 48 (96.0) | 50 (100.0) |  | 46 (90.2) | 48 (96.0) | 46 (92.0) | 48 (96.0) |
| Other | - | - | - | - |  | 2 (3.92) | - | - | 1 (2.0) |
| **Fruit washing before eating** |  |  |  |  |  |  |  |  |  |
| Nothing | - | 1 (2.0) | - | - |  | - | - | - | - |
| Wash with water | 49 (98.0) | 49 (98.0) | 50 (100.0) | 49 (98.0) |  | 44 (86.3) | 40 (80.0) | 43 (86.0) | 47 (94.0) |
| Clean with hands | - | - | - | 1 (2.0) |  | - | - | - | - |
| Other | 1 (2.0) | - | - | - |  | 7 (13.7) | 10 (20.0) | 7 (14.0) |  |
| **Water to drink** |  |  |  |  |  |  |  |  |  |
| Every kind | - | 2 (4.0) | - | 1 (2.0) |  | - | - | - | - |
| Spring water | - | 8 (16.0) | 17 (34.0) | 2 (4.0) |  | 1 (1.9) | 2 (4.0) | 2 (4.0) | 7 (14.0) |
| Pump water | 50 (100.0) | 40 (80.0) | 32 (64.0) | 46 (92.0) |  | 47 (92.1) | 48 (96.0) | 47 (94.0) | 40 (80.0) |
| Others | - | - | 1 (2.0) | 1 (2.0) |  | 3 (5.9) | - | 1 (2.0) | 3 (6.0) |
| **Cover food** |  |  |  |  |  |  |  |  |  |
| Not cool | - | - | - | 1 (2.0) |  | - | - | - | - |
| Avoid dirty | 41 (82.0) | 47 (94.0) | 36 (72.0) | 40 (80.0) |  | 47 (92.2) | 38 (76.0) | 40 (80.0) | 43 (86.0) |
| Avoid flies | 4 (8.0) | 1 (2.0) | 7 (14.0) | 3 (6.0) |  | 2 (3.9) | 6 (12.0) | 2 (4.0) | 1 (2.0) |
| Avoid sand | - | - | - | 6 (12.0) |  | - | - | - | 1 (2.0) |
| Others | 5 (10.0) | 2 (4.0) | 7 (14.0) | - |  | 2 (3.9) | 6 (12.0) | 8 (16.0) | 5 (10.0) |
| **Wearing shoes** |  |  |  |  |  |  |  |  |  |
| Not to hurt | - | 2 (4.0) | 4 (8.0) | 1 (2.0) |  | 4 (7.8) | 4 (8.0) | 1 (2.0) | - |
| Avoid worms | 40 (80.0) | 39 (78.0) | 34 (68.0) | 29 (58.0) |  | 47 (92.2) | 40 (80.0) | 42 (84.0) | 45 (90.0) |
| Do not walk in the dirt | 1 (2.0) | 1 (2.0) | 2 (4.0) | 1 (2.0) |  | - | 3 (6.0) | - | 1 (2.0) |
| Other | 9 ((18.0) | 8 (16.0) | 10 (20.0) | 19 (38.0) |  | - | 3 (6.0) | 7 (14.0) | 4 (8.0) |
| **Why not bath in the river** |  |  |  |  |  |  |  |  |  |
| Keep warm | - | - | - | - |  | - | - | - | - |
| Avoid schistosomiasis | 10 (20.0) | 25 (50.0) | 13 (26.0) | 13 (26.0) |  | 30 (58.8) | 32 (64.0) | 33 (66.0) | 27 (54.0) |
| It’s drinking water | 1 (2.0) | - | 1 (2.0) | - |  | 1 (2.0) | - | 1 (2.0) | 2 (4.0) |
| Avoid drowning | 1 (2.0) | - | 1 (2.0) | - |  | 1 (2.0) | - | 1 (2.0) | - |
| Other | 38 (76.0) | 25 (50.0) | 35 (70.0) | 37 (74.0) |  | 19 (37.2) | 17 (34.0) | 16 (32.0) | 20 (40.0) |
