# Supplementary material for: “Koko et les lunettes magiques”: An educational entertainment tool to prevent parasitic worms and diarrheal diseases in Côte d’Ivoire
Source: PLoS Negl Trop Dis. 2017 Sep 21;11(9):e0005839. doi: 10.1371/journal.pntd.0005839 (PMC5630154; doi:10.1371/journal.pntd.0005839)
Supplement: S3 Table — (DOCX) [file pntd.0005839.s003.docx]

**S3 Table.** Knowledge of symptoms caused by worms following the screening of “Koko et les lunettes magiques” during the pilot testing in eight schools of south-central (Tiassalé) and western (Man) Côte d’Ivoire.

| **Variable** | **Tiassalé** |  |  |  |  | **Man** |  |  |  |
| --- | --- | --- | --- | --- | --- | --- | --- | --- | --- |
|  | **Binao**  **(%)** | **Boussoué**  **(%)** | **Niamoué**  **(%)** | **Tiassalékro (%)** |  | **Dompleu**  **(%)** | **Kogouin**  **(%)** | **Krikouma**  **(%)** | **Zê**  **(%)** |
| **Fatigue** |  |  |  |  |  |  |  |  |  |
| Yes | 14 (28.0) | 23 (46.0) | 15 (30.0) | 11 (22.0) |  | 44 (86.3) | 46 (92.0) | 38 (76.0) | 43 (86.0) |
| No | 36 (72.0) | 27 (54.0) | 35 (70.0) | 39 (78.0) |  | 7 (13.7) | 4 (8.0) | 12 (24.0) | 7 (14.0) |
| **Lack of concentration** |  |  |  |  |  |  |  |  |  |
| Yes | 14 (28.0) | 19 (38.0) | 13 (26.0) | 15 (30.0) |  | 35 (68.6) | 39 (78.0) | 28 (56.0) | 36 (72.0) |
| No | 36 (72.0) | 31 (62.0) | 37 (74.0) | 35 (70.0) |  | 16 (31.4) | 11 (22.0) | 22 (44.0) | 14 (28.0) |
| **Diarrhea** |  |  |  |  |  |  |  |  |  |
| Yes | 33 (66.0) | 33 (66.0) | 34 (68.0) | 20 (40.0) |  | 33 (64.7) | 43 (86.0) | 38 (76.0) | 42 (84.0) |
| No | 17 (34.0) | 17 (34.0) | 16 (32.0) | 30 (60.0) |  | 18 (35.3) | 7 (14.0) | 12 (24.0) | 8 (16.0) |
| **Stunting** |  |  |  |  |  |  |  |  |  |
| Yes | 14 (28.0) | 14 (28.0) | 10 (20.0) | 5 (10.0) |  | 39 (76.5) | 40 (80.0) | 34 (68.0) | 35 (70.0) |
| No | 36 (72.0) | 36 (72.0) | 40 (80.0) | 45 (90.0) |  | 12 (23.5) | 10 (20.0) | 16 (32.0) | 15 (30.0) |
| **Stomach aches** |  |  |  |  |  |  |  |  |  |
| Yes | 26 (52.0) | 33 (66.0) | 33 (66.0) | 33 (66.0) |  | 45 (88.2) | 47 (94.0) | 50 (100.0) | 49 (98.0) |
| No | 24 (48.0) | 17 (34.0) | 17 (34.0) | 17 (34.0) |  | 6 (11.8) | 3 (6.0) | - | 1 (2.0) |
| **Lack of appetite** |  |  |  |  |  |  |  |  |  |
| Yes | 18 (36.0) | 20 (40.0) | 18 (36.0) | 17 (34.0) |  | 32 (62.7) | 36 (72.0) | 32 (64.0) | 30 (60.0) |
| No | 32 (64.0) | 30 (60.0) | 32 (64.0) | 33 (66.0) |  | 19 (37.3) | 14 (28.0) | 18 (36.0) | 20 (40.0) |
| **Do not think well** |  |  |  |  |  |  |  |  |  |
| Yes | 12 (24.0) | 18 (36.0) | 11 (22.0) | 9 (18.0) |  | 33 (64.7) | 38 (76.0) | 21 (42.0) | 30 (60.0) |
| No | 38 (76.0) | 32 (64.0) | 39 (78.0) | 41 (82.0) |  | 18 (35.3) | 12 (24.0) | 29 (58.0) | 20 (40.0) |
| **Don’t know** |  |  |  |  |  |  |  |  |  |
| Yes | - | 2 (4.0) | 3 (6.0) | 1 (2.0) |  | 1 (2.0) | 2 (4.0) | - | - |
| No | 50 (100.0) | 48 (96.0) | 47 (94.0) | 49 (98.0) |  | 50 (98.0) | 48 (96.0) | 50 (100.0) | 50 (100.0) |
|  |  |  |  |  |  |  |  |  |  |
